# Case of Arrested Muscular Development

**Published:** 1850-08

**Authors:** P. K. Huntington

**Affiliations:** Perry, Wyoming County, New York


*Case of Arrested Muscular Development.* By P. K. Huntington, M. D., of Perry, Wyoming County, New York. (Communicated by Prof. J. K. Mitchell.)

Mr. Benedict, a young man, aged 22 years, of good habits, has had, without any apparent cause, for the last eight years, no developement whatever of the muscles of the thighs and pelvis, and also of the arms, while those of the leg, fore-arm, foot and hand, and also of the back, are fully developed. The gastrocnemii are very large indeed, resembling much those of an opera dancer, while the muscles of the thigh, including the glutei, are flaccid and shrunken, resembling those we find in the limbs of a person in the last stages of phthisis. The contrast between the arm and fore-arm is not quite so striking as that of the corresponding parts of the lower extremities, yet it is very apparent to any observer.

He complains of no pain or inconvenience whatever, and suffers only from the weakness which necessarily attends such debility of the muscles.

Whenever he rises from a sitting posture to a standing one, it is done by the assistance of the upper extremities, and a sort of springing motion. He cannot step up a common stair without a very great effort, accompanied also with a sudden spring. To raise himself from the stooping posture is impossible without extrinsic mechanical aid.

There is no apparent difference in the sides of his body; both seem affected alike.

He imagines that the muscles affected are less in size than they were eight years ago, but whether this is really the case, or whether it has been merely an arrest in the developement, while the rest of the body has been developed naturally, is as yet a question unanswered.

I have searched the works to which I have access to find an analogous case, but in vain, for I can find nothing which even approximates it.

I would advise him to visit your city, were I satisfied that any medical aid would benefit him.

With this concise description, therefore, I wish to submit the case to you, asking your opinion in regard to the propriety and probable success of medication.

